# 

**Published:** 1903-08

**Authors:** 


### CONTRIBUTORS TO NEW SERIES VOLUME XLIII.

### (Whole Number Volume LIX.)

### AUGUST, 1903—JULY 1904.

---

| | |
|---|---|
| Bagley, Thomas | Buffalo |
| Beahan, A. L. | Canandaigua |
| Bishop, Louis Faugeres | New York |
| Brown, C. W. M. | Elmira |
| Burrows, Lorenzo, Jr. | Buffalo |
| Cheesman, William S. | Auburn |
| Clark, Clarence G. | New York |
| Clark, Joseph C. | Olean |
| Clinton, Marshall | Buffalo |
| Clowes, G. H. A. | Buffalo |
| Congdon, Charles E. | Buffalo |
| Curtis, B. Farquhar | New York |
| Daggett, Byron H. | Buffalo |
| Dignen, William E. | Buffalo |
| Dowd, J. Henry | Buffalo |
| Frye, Maud J. | Buffalo |
| Glenny, W. Harry | Buffalo |
| Goelet, Augustin H | New York |
| Gravel, Louis J. | Montreal |
| Griffith, Jefferson D. | Kansas City |
| Hanley, Lawrence G. | Buffalo |
| Harrington, D. W | Buffalo |
| Hayd, Herman E. | Buffalo |
| Heffron, John L. | Syracuse |
| Higgins, Frank W. | Cortland |
| Hoffmann, Frank F. | Chicago |
| Hopkins, Henry Reed | Buffalo |
| Howe, Lucien | Buffalo |
| Hubbell, Alvin A. | Buffalo |
| Jack, George N. | Buffalo |
| Jewett, Charles Sherman | Buffalo |
| Jones, William B. | Rochester |
| Krauss, William C. | Buffalo |
| LeBreton, Prescott | Buffalo |
| Lewis, Bransford | St. Louis |
| Lewis, F. Park | Buffalo |
| Lewis, G. Griffin | Syracuse |
| Loveland, Bradford C. | Syracus |

McCormack, J. N. . . . Bowling Green, Ky.
McMurtry, Lewis S . . . Louisville
Mann, Matthew D. . . . Buffalo
Millener, Frederick Hoyer . . . Buffalo
Miller, John A. . . . Buffalo
Park, Roswell . . . Buffalo
Parmenter, John . . . Buffalo
Potter, William Warren . . . Buffalo
Putnam, James Wright . . . Buffalo
Renner, W. Scott . . . Buffalo
Ricketts, B. Merrill . . . Cincinnati
Satterlee, Richard H . . . Buffalo
Smith, Chauncey Pelton . . . Buffalo
Smith, Eugene A. . . . Buffalo
Smith, Edward T. . . . Buffalo
Smith, Frank Hyatt . . . Buffalo
Stockton, Charles G . . . Buffalo
Toepfer, A . . . Jersey City
Urban, Adolph H . . . Buffalo
Van Duyn, E. S . . . Syracuse
Van Fleet, Frank . . . New York
Wallace, W. L . . . Syracuse
Wende, Ernest . . . Buffalo
Wende, Grover W . . . Buffalo
Wey, Hamilton D . . . Elmira
Wheeler, David E . . . Buffalo
Wilson, Nelson W . . . Buffalo

Alumni Association, University of Buffalo
Buffalo Academy of Medicine.
Lake Keuka Medical and Surgical Association.
Medical Association of Central New York.
Medical Society of the County of Erie.
Medical Society of the State of New York.
New York Odontological Society.
New York State Medical Association.
Roswell Park Medical Club.

### BOOKS RECEIVED.

Gynecology. A Textbook for Students and a Guide for Practitioners. By William R. Pryor, M. D., Professor of Gynecology in the New York Polyclinic Medical School; Attending Gynecologist to New York Polyclinic Hospital; Consulting Gynecologist to St. Vincent's, New York City and St. Elizabeth's Hospitals. Octavo, pp. 396, with 163 illustrations in the text. New York and London. D. Appleton & Company. 1903. (Price, $3.50.)

The Practical Medicine Series of Year Books. Ten volumes. Issued under the general editorial charge of Gustavus P. Head, M. D., Professor of Laryngology and Rhinology in the Chicago Post-Graduate Medical School. Vol. VI. General Medicine. Edited by Frank Billings, M.S., M. D. Head of the Medical Department and Dean of the Faculty of Rush Medical College, Chicago. And J. H. Salisbury, M. D., Professor of Medicine, Chicago Clinical School. Duodecimo, pp. 316. Chicago: The Year Book Publishers. 1903. (Price, $1.50; entire series, $7.50.)

Medical Reports of the Sheppard and Enoch Pratt Hospital. Vol. I., No. 1. Baltimore, Md. 1903.

A Reference Handbook of the Medical Sciences by various writers. New edition. Edited by Albert H. Buck, M. D. Volume VI. Imp. Quarto. 1012 pages. 764 engravings. Nine full-page plates, in black and colors. New York: William Wood & Company. 1903.

First Principles of Otology. A Textbook for Medical Students. By Albert H. Buck, M. D. Clinical Professor of the Diseases of the Ear, College of Physicians and Surgeons, Medical Department of Columbia University, New York. Duodecimo, pp. 227. Second edition. Illustrated. New York: William Wood & Company. 1903. (Price, $1.50.)

Transactions of the Southern Surgical and Gynecological Association. Vol. XV. Annual meeting, held at Cincinnati, O., November 11, 12 and 13, 1902. W. D. Haggard, M. D., Secretary. Philadelphia: Wm. J. Dornan, Printer. 1903.

A Treatise on Organic Nervous Diseases, by M. Allen Starr, M. D., Ph.D., LL.D., Professor of Diseases of the Mind and Nervous System in the College of Physicians and Surgeons (Medical Department of Columbia University), New York; Consulting Neurologist to the Presbyterian and St. Vincent's Hospitals, St. Mary's Free Hospital for Children, etc. In one octavo volume of 751 pages, with 275 engravings and 26 plates in colors and monochrome. Lea Brothers & Co., New York and Philadelphia. 1903. (Cloth, $6.00; leather, $7.00 net.)

Lea's Medical Epitome Series. Medical Jurisprudence. A Manual for Students and Practitioners. By Edwin Welles Dwight, M. D., Instructor in Legal Medicine, Harvard University. Series edited by V. C. Pedersen, A.M., M.D., Instructor in Surgery at the New York Polyclinic Medical School and Hospital. Duodecimo, pp. 249. Philadelphia and New York: Lea Brothers & Co. 1903.

International Clinics. A Quarterly of Illustrated Clinical Lectures and especially prepared original Articles on Treatment, Medicine, Surgery, Neurology, Pediatrics, Obstetrics, Gynecology, Orthopedics, Pathology, Dermatology, Ophthalmology, Otology, Rhinology, Laryngology, Hygiene, and other topics of interest to Students and Practitioners, by leading members of the medical profession throughout the world. Edited by A. O. J. Kelly, A.M., M. D., Philadelphia, with the collaboration of Wm. Osler, M. D., Baltimore; John H. Musser, M. D., Philadelphia; Jas. Stewart, M. D., Montreal; John B. Murphy, M. D., Chicago; Thomas M. Rotch, M. D., Boston; John G. Clark, M. D., Philadelphia; James J. Walsh, M. D., New York; J. W. Ballantyne, M. D., Edinburgh; John Harold, M. D., London; Edmund Landolt, M. D., Paris, and Richard Kretz, M. D., Vienna. With regular correspondents in Montreal, London, Paris, Berlin, Vienna, Leipsic, Brussels and Carlsbad. Volume II. Thirteenth series. 1903. Philadelphia: J. B. Lippincott Company. 1903. (Cloth, $2.00.)

Lea's Medical Epitome Series. Microscopy and Bacteriology. A Manual for Students and Practitioners. By P. E. Archinard, A.M., M. D., Demonstrator of Microscopy and Bacteriology, Tulane University of Louisiana, Medical Department. Duodecimo, pp. 210. Illustrated with 74 engravings. Series edited by V. C. Pedersen, A.M., M. D., Instructor in Surgery at the New York Polyclinic Medical School and Hospital. Lea Brothers & Co., Philadelphia and New York. 1903.

A Thesaurus of Medical Words and Phrases. By Wilfred M. Barton, M. D., Assistant to Professor of Materia Medica and Therapeutics, and Lecturer on Pharmacy, Georgetown University, Washington, D. C.; and Walter A. Wells, M. D., Demonstrator of Laryngology and Rhinology, Georgetown University, Washington, D. C. Handsome octavo of 534 pages. Philadelphia, New York, London: W. B. Saunders & Company. 1903. (Flexible leather, $2.50 net; with thumb index, $3.00 net.)

